# Moral Decision-Making, Stress, and Social Cognition in Frontline Workers vs. Population Groups During the COVID-19 Pandemic: An Explorative Study

**DOI:** 10.3389/fpsyg.2020.588159

**Published:** 2020-11-19

**Authors:** Monica Mazza, Margherita Attanasio, Maria Chiara Pino, Francesco Masedu, Sergio Tiberti, Michela Sarlo, Marco Valenti

**Affiliations:** ^1^Department of Applied Clinical Sciences and Biotechnology, University of L’Aquila, L’Aquila, Italy; ^2^Regional Centre for Autism, Abruzzo Region Health System, L’Aquila, Italy; ^3^Department of Communication Sciences, Humanities and International Studies, University of Urbino Carlo Bo, Urbino, Italy

**Keywords:** COVID-19 pandemic, moral decision-making, moral dilemmas, stress, empathy, Theory of Mind, frontline workers

## Abstract

On March 9, 2020, Italy has gone into “lockdown” because of COVID-19 pandemic, with a national quarantine. All non-essential working activities and schools of all levels have been temporarily closed: consequently, the entire population have been forced to dramatically change their daily habits. The pandemic raised important psychological, moral, social, and economic issues. Our research focused on the moral decision-making of people during an emergency. This paper reports two studies. The aim of Study 1 was to evaluate moral decision-making, level of perceived stress, ability of mentalizing and empathy in university students and Italian workers. 224 front-line workers (FLW), 413 second-line workers (SLW), and 663 university students (US), during Italian Phase 1 of lockdown, completed an online questionnaire. The results of Study 1 showed that participants in the FLW group are more likely to choose utilitarian solutions and judge as morally acceptable actions finalized to saving lives of more people if this requires sacrificing a low number of individuals. At the same time, decision-making was experienced as less unpleasant and less arousing with respect to the other two groups, demonstrating a greater ability to keep emotional control under pressure. In Study 2, we compared the same variables used in Study 1, selecting two professional categories from the FLW group engaged in emergency during COVID-19, namely healthcare providers (*n* = 82) and public safety personnel (n = 117). Our results showed that healthcare providers were more stressed and emotionally involved than public safety personnel, with higher empathic concern and arousal in moral decision-making. We suggest it is essential providing immediate psychological support and monitoring physical and emotional well-being for workers in the front-line during emergencies like the COVID-19 pandemic, in order to prevent experiences of moral distress or mental health problems.

## Introduction

In March 2020, the outbreak of Coronavirus disease (COVID-19) placed Italy in front of unprecedented health, social, economic, and political challenges. All non-essential working activities and schools of all levels were temporarily closed: consequently, the entire population have been forced to dramatically change their daily habits.

Many Italian university students remained away from their hometown during the lockdown and some had to face the postponement of exams or degrees and the uncertainty about future. Italian workers have suffered changes in their work routine: some people adopted remote or smart working (others continued to work in critical conditions by adopting safety measures that were not always adequate, while others lost their jobs or salary. At all levels, the challenge was between economic safeguard and population health.

The COVID-19 pandemic has raised important moral and ethical issues at different levels, i.e., respect quarantine rules and sacrifice for collective well-being; saving economy or human lives; choosing patients to be treated first; continuing work activities and putting the health of loved ones at risk; deciding to go back to hometown with the risk of spreading infection. Particularly during a pandemic, moral decision-making involves uncertainty (Van Bavel et al., 2020); furthermore, moral dilemmas and moral distress are often inevitable (Dunham et al., 2020). A moral dilemma is a problematic situation that involves a conflict between two mutually exclusive alternatives, both implying negative and undesirable consequences (Sinnott-Armstrong, 1987; Tasso et al., 2017; Palmiotti et al., 2020). These are situations in which the individual is faced with two moral principles, in opposition to each other, which imply making a decision: maximizing the common good according to a cost-benefit analysis (utilitarian resolution) or deciding for the unconditional respect for a moral rule, regardless of the consequences (deontological resolution). Moral distress occurs when individuals know what is the ethically appropriate choice but they are unable to do it due to external or internal restrictions (McCarthy and Deady, 2008; Epstein and Hamric, 2009; Dean et al., 2020; Dunham et al., 2020). During an emergency like a pandemic, some decisions are made under stress and several studies showed that stress can influence moral decision-making (Lützen et al., 2010; Starcke et al., 2011; Starcke and Brand, 2012; Youssef et al., 2012; Romero-Rivas and Rodríguez-Cuadrado, 2020). As pointed out by Francis and McNabb (2020), the COVID-19 pandemic caused radical changes in social, community, health, and political practices that could affect what is considered right or wrong and moral principles underlying decision-making processes. Moreover, public messages inspired by moral principles have increased considerably during pandemic (Everett et al., 2020; Francis and McNabb, 2020). These public messages from government institutions, celebrities and health officials, urged citizens to adopt certain behaviors as moral imperatives referring, for example, to utilitarian, virtue-based or deontological moral theories (Mill, 1863; Singer, 1972; Hursthouse, 1999; Scanlon, 2003; Brooks et al., 2020; Everett et al., 2020). Moral judgment and social cognition abilities, in particular Theory of Mind (ToM) and empathy, are closely interrelated (Hoffman, 1991; Singer et al., 2004; Forbes and Grafman, 2010; Young et al., 2010; Baez et al., 2017; Del Casale et al., 2017; Eres et al., 2018; Schaller et al., 2019). According to the dual-process model (Greene et al., 2001, 2004, 2008), moral decision-making involves cognitive and affective processes to conflict each other. Cognitive processes, which are relatively slow and based on deliberative reasoning, support utilitarian resolutions and involve the activation of brain areas associated with working memory, problem solving, abstract thinking and cognitive control. On the contrary, affective processes, which are fast and automatic, operate independently from cognitive resources, favor deontological solutions and involve the activation of brain areas associated with emotional processing and social cognition (Greene et al., 2001, 2004; Sarlo et al., 2012; Patil et al., 2020). These evidences support the assumption that moral decision-making involves social cognitive processes (Moll et al., 2002a; Young et al., 2007; Moran et al., 2011; Bzdok et al., 2012).

Moral judgment requires both ToM – i.e., the ability to infer mental states like other people’s intentions, beliefs, emotions, and desires (Cushman, 2008; Young and Saxe, 2009; Fu et al., 2014; Happé and Frith, 2014; Sodian et al., 2016; Baez et al., 2017) – and empathy – i.e., the capacity to share and understand the subjective experience of others about oneself (Decety, 2011; Baez et al., 2017).

Theory of Mind and empathy help us to judge the social consequences of behaviors (Moll et al., 2002a, b; Adolphs, 2003; Greene et al., 2004; Eslinger et al., 2009; Reniers et al., 2012), encourage prosocial behavior (Eisenberg, 2000; Reniers et al., 2012; Sarlo et al., 2014), support the appropriate responses to the perceived feelings of people around us (Baron-Cohen et al., 2003;, Shamay-Tsoory et al. 2003; Vreeke and van der Mark, 2003; Reniers et al., 2012), and prevent to harming others (Batson et al., 1991; Eisenberg, 2000; Sarlo et al., 2014).

Some recent studies examined empathy (Jordan et al., 2020; Oosterhoffand Palmer, 2020; Pfattheicher et al., 2020), psychological consequences (Cao et al., 2020; Elmer et al., 2020; Li et al., 2020; Oosterhoffand Palmer, 2020; Qiu et al., 2020; Wang et al., 2020) and moral decision-making (Francis and McNabb, 2020; Romero-Rivas and Rodríguez-Cuadrado, 2020) in people during COVID-19 pandemic. To our knowledge, there are no studies that focused on specific categories of individuals that have undergone different changes in their lives during pandemic.

## Objective

The primary aim of our study, exposed in Study 1, was to evaluate moral decision-making, stress, and empathy in Italian workers and university students. In Study 2, we compared two categories of front-line workers that, during the pandemic, worked in critical conditions and immediate management of the emergency, i.e., healthcare providers (HP) and public safety personnel (PSP).

## Materials and Methods of Study 1 and 2

### Procedure

Data of both studies were collected between March 30 and May 4, 2020 (during Phase One of Italy’s coronavirus lockdown) using an on-line questionnaire created on the platform Google Form. The questionnaire investigated key demographic variables, workplace characteristics, such as being a front-line or second-line worker during COVID-19, and tested several individual characteristics: moral decision-making, stress, and social abilities such as empathy and ToM. The duration of the entire questionnaire was about 30 min. On-line informed consent was obtained from the participants in accordance with the Declaration of Helsinki (World Medical Association, 2013).

### Measures

The online questionnaire used for both studies included the following instruments:

*Set of Moral Dilemmas* (Lotto et al., 2014). We selected 25 moral dilemmas by a standardized set of Lotto et al. (2014) in order to evaluate the moral decision-making. Specifically, we used 10 *Incidental dilemmas*, which described killing one or two individuals as an expected but unintended consequence of saving other people; 10 *Instrumental dilemmas*, which described killing one or two individuals as a means to save other people. Each of these two types of dilemmas was varied for risk involvement (Lotto et al., 2014). Thus, in 5 dilemmas killing one or two individuals saves one’s own and other people’s lives (*Self-involvement dilemmas*), whereas in five dilemmas killing one or two individuals saves only other people’s lives (*Other-involvement dilemmas*). Each class of dilemmas were matched for the number of victims. We included also five “filler dilemmas” in which there were no deaths but only moral issues such as being dishonest or lying, in order to avoid automaticity in responding to conceptually similar issues. Each dilemma was presented as a text that described the scenario where some kind of danger was going to cause the death of a group of people. Each scenario ended with the proposal of a utilitarian resolution and participants were asked to indicated whether they would do the suggested action by choosing between “yes” or “no” (*Would you do it?)*. Immediately after their decision, the participants were asked to judge how morally acceptable the proposed resolution was (*How morally acceptable is the proposed action?*) on an 8-point scale (0 = not at all, 7 = completely). Finally, participants were asked to rate their emotional state during decision-making (*How did you feel while making the decision?*) using the Self-Assessment Manikin (SAM; Bradley and Lang, 1994) including the valence scale ranging from 1 (extreme unpleasantness) to 9 (extreme pleasantness) points and the arousal scale ranging from 1 (extreme calm) to 9 (extreme activation) points.

*Perceived Stress Scale* (PSS-10) was used to measure the degree to which the participants appraise events as stressful during the past month (Cohen et al., 1983). It comprises 10 items that allow five responses on a Likert scale: never (0), almost never (1), once in a while (2), often (3), and very often (4). Six items of the PSS-10 are considered negative (item 1, 2, 3, 6, 9, and 10), which assess the level of distress; the other four are positive (item 4, 5, 7, and 8) and reflect the perception of a person’s ability to cope with the stressors. The positive items were reversely coded when calculating the total score of the PSS-10. The total score of the PSS-10 ranges from 0 to 40, with higher scores indicating more stress (Sun et al., 2019).

*Interpersonal Reactivity Index* (IRI): the IRI, which Davis (1983) developed, is the most frequently used self-administered instrument to assess the different components of empathy. The IRI includes four sub-scales: fantasy (FS), perspective taking (PT), personal distress (PD), and empathic concern (EC). The FS sub-scale evaluates the tendency of the individual to identify him or herself with fictitious personages, such characters from books, films, or video games (e.g., “When I am reading an interesting story or novel, I imagine how I would feel if the events in the story were happening to me”). The PT sub-scale evaluates the tendency of an individual to spontaneously adopt the psychological point-of-view of another person (e.g., “I sometimes find it difficult to see things from the other guy’s point of view”). The PD sub-scale evaluates discomfort in reaction to other’s people emotions (e.g., “When I see someone get hurt, I tend to remain calm”). The EC sub-scale refers to feelings of compassion, tenderness, and concern for other people (e.g., “When a friend tells me about his good fortune, I feel genuinely happy for him”).

*Advanced Theory of Mind Task* (A-ToM; Blair and Cipolotti, 2000) it is the Italian adaptation version of the ToM’ s task firstly proposed by Happé (1994). It consists of 13 stories which describe real events; for a correct interpretation the task requires subjects to go beyond the literal meaning of the text and make an inference about the protagonist’s mental state. The 13 stories were made not to be ambiguous, so that each story could have a single interpretation. Each story presents different types of mental state attribution: Pretend, Persuade, Joke, Lie, White Lie, Misunderstanding, Irony, Double Bluff and Sarcasm. Each story is followed by two questions: one comprehension question (e.g., “Was it true, what X said?”) and a justification question (e.g., “Why did X say that?”). Each item could be assigned a score of 1 when comprehension and justification questions are answered correctly, otherwise, a score of 0 is assigned, thus the total score could range between 0 and 13. Happé (1994) used the term “advanced” to refer to a story that contains the comprehension question, where the key questions in the task concerned a character’s mental states (the experimental condition) to explain the cause about his/her behavior (Pino and Mazza, 2016).

## Study 1

The aim of study 1 was to compare the moral decision-making, level of perceived stress, the ability of mentalizing, and empathy in front-line workers (FLW), second-line workers (SLW), and university students (US) during COVID-19 pandemic. As pointed out by Francis and McNabb (2020), moral judgment can be influenced by specific features of the situation and characteristics of decision-makers, such as mood.

These three categories of individuals had a different level of exposure risk during the pandemic. Occupations in sectors that were fundamental during the epidemic, such as healthy industry or food industry, were more directly exposed to infection than who work remotely, such as in public administration or education sectors (Barbieri et al., 2020). Williamson et al. (2020) suggested that front-line key workers (e.g., healthcare providers and emergency first responders), but also workers in essential sectors (e.g., supermarket workers or delivery drivers) may be especially exposed to experiencing moral injuring during a pandemic due to a lack of adequate resources, clear guidance, specific training, or psychological support. On the other hand, SLW had to reorganize their work routines and were exposed to greater social isolation or, else, to forced proximity with immediate family (Van Bavel et al., 2020). These drastic changes also affected US (Cao et al., 2020; Elmer et al., 2020). The COVID-19 pandemic may have influenced socio-emotional and psychological aspects in the three groups in different ways.

### Participants

In Study 1, 1300 Italian people answered our online questionnaire. Among these, 8.6% lived in northern regions, 52% lived in southern regions and 39.4% lived in central regions.

The age range of the entire sample went from 18 to 66 years (for details see Table 1).

**TABLE 1:** Differences among front-line workers (FLW), second-line workers (SLW), and university students (US) for demographic data.

	FLW (*N* = 224)	SLW (*N* = 413)	US (*N* = 663)	*F (df)*	*p*	η${}_{p}^{2}$
Mean Chronological Age in years (SD)	38.39 (10.89)	38.69 (12.93)	22.94 (4.12)	483.78 (2.13)	0.0001*	0.43
Mean Education in years (SD)	15.11 (3.56)	15.98 (3.65)	13.43 (1.39)	117.95 (2.13)	0.0001	0.15
Gender (M; F)	146; 78	125; 288	106; 557	197.236 (2)**	0.0001**	–

*There were no significant differences between FLW and SLW. **Chi-square test.

Participants were divided into three groups, based on COVID-19 emergency: 224 FLW, 413 SLW, and 663 US. The FLW are employees who provide an essential service or key public service (e.g., health care workers, public safety workers, supermarket workers, firefighters). The SLW are workers who, during COVID-19 emergency, shifted to remote working or for whom contact with other people was minimized (e.g., teachers and professors, computer scientists, employees in public administration). Finally, the US group in the period of quarantine experienced a situation of uncertainty and concern for their university career and their future in general.

### Statistical Analysis

We performed a one-way analysis of variance (ANOVA) to evaluate differences among the three groups (FLW, SLW, and US) in the sociodemographic data.

Regarding the moral decision-making task, we calculated the following variables for each participant and each dilemma type:

(a) the proportion of utilitarian choices was calculated by dividing the number of “yes” answers by the total number of responses to each dilemma type;
(b) the mean ratings of moral acceptability;
(c) the mean ratings of valence;
(d) the mean ratings of arousal.

We performed four separate 3 × 2 × 2 repeated-measures ANOVAs on the proportion of utilitarian choices, mean ratings of moral acceptability, means ratings of valence and mean ratings of arousal. For each of these variables, we considered the *Group* (FLW, SLW, and US) as a between-subject factor, and *Type of Dilemma* (Incidental vs Instrumental) and *Risk-Involvement* (Self vs Other-involvement) as within-subject factors. Fisher’s Least Significant Difference (LSD) post-hoc comparisons were conducted on significant main effects and interactions.

Finally, we performed a one-way ANOVA to evaluate the differences among the three groups in the mean scores of PSS, A-ToM, and all the subscales of IRI. Fisher’s LSD post-hoc comparisons were conducted on significant main effects.

The analyses were performed using IBM SPSS Statistics (IBM Corp., 2011).

## Results

### Moral Decision-Making Task

#### Proportion of Utilitarian Choices

The *Group* main effect was significant (*F*_2,1297_ = 3.48, *p* = 0.03, η${}_{p}^{2}$ = 0.005), with participants in the FLW group more inclined to sacrifice one or two persons to save a larger number of lives as compared to participants in the SLW group (*p* = 0.014) and the US group (*p* = 0.017). We found no significant differences between the SLW group and the US group.

**FIGURE 1:**
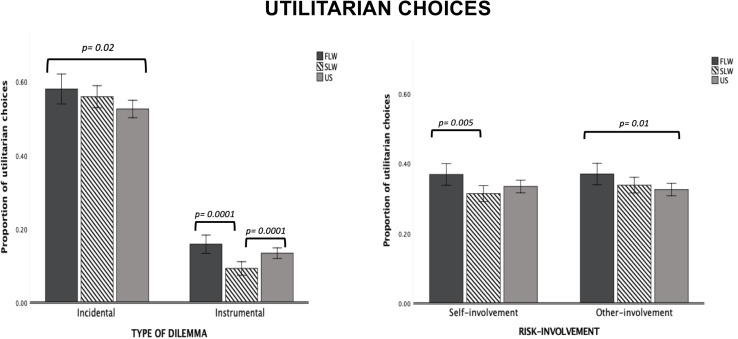
Bar graphs depict the significant Type of Dilemma X Group interaction and Risk-Involvement X Group interaction for proportion of utilitarian choices in the moral decision-making task. Fisher’s LSD post-hoc comparisons were conducted on significant main effects and interactions. In the figure, statistically significant differences among groups are indicated with *p* values. Error bars indicate the standard errors. FLW, front-line workers; SLW, second-line workers; US, university students.

*Type of Dilemma* (*F*_1,1297_ = 2591.27, *p* = 0.0001, η${}_{p}^{2}$ = 0.66), but not Risk-involvement, was significant, with Incidental dilemmas receiving more utilitarian responses than Instrumental dilemmas (*p* = 0.0001). We found a significant *Type of Dilemma* × *Group* interaction (*F*_2,1297_ = 9.448, *p* = 0.0001, η${}_{p}^{2}$ = 0.01). Post-hoc comparisons showed that the FLW group was more likely to accept utilitarian choices than the US group on Incidental dilemmas (*p* = 0.02); no significant differences were found between the SLW group and the other two groups. On Instrumental dilemmas, the SLW group was more likely to reject utilitarian choices than the FLW group (*p* = 0.0001) and the US group (*p* = 0.0001; see Figure 1); no significant differences were found between the US group and the FLW group. We also found a significant *Risk-involvement* × *Group* interaction (*F*_2,1297_ = 4.088, *p* = 0.01, η${}_{p}^{2}$ = 0.006). Post-hoc tests indicated that, on dilemmas with self-involvement, the FLW group provided a greater proportion of utilitarian choices compared to the SLW group (*p* = 0.005); no significant differences were found between the US group and the other two groups. On dilemmas with other-involvement, the FLW group provided a greater proportion of utilitarian choices than the US group (*p* = 0.01*;* see Figure 1); no significant differences were found between the SLW group and the other two groups.

#### Moral Judgment

*Type of Dilemma* (*F*_1,1297_ = 557.9, *p* = 0.0001, η${}_{p}^{2}$ = 0.30) and *Risk- involvement* (*F*_1,1297_ = 19.67, *p* = 0.0001, η${}_{p}^{2}$ = 0.01) main effects were both significant, with the utilitarian choices on Incidental dilemmas and Other-involvement dilemmas judged as more morally acceptable compared to Instrumental dilemmas and Self-involvement dilemmas, respectively (*p* = 0.0001 for each comparison). We also found a significant *Type of Dilemma* × *Risk-involvement* (*F*_1,1297_ = 25.11, *p* = 0.0001, η${}_{p}^{2}$ = 0.02) interaction. Post-hoc tests showed that the *Risk-involvement* effect was significant only for Incidental dilemmas (*p* = 0.0001). Specifically, our participants judged Incidental Other-involvement dilemmas were judged as more morally acceptable than Incidental Self-involvement dilemmas (*p* = 0.0001).

The significant *Group* main effect (*F*_2,1297_ = 33.13, *p* = 0.0001, η${}_{p}^{2}$ = 0.05) showed that the FLW group judged the utilitarian choices as more morally acceptable than the SLW group and the US group (*p* = 0.0001 for each comparison). We found no significant differences between the SLW group and the US group.

**FIGURE 2:**
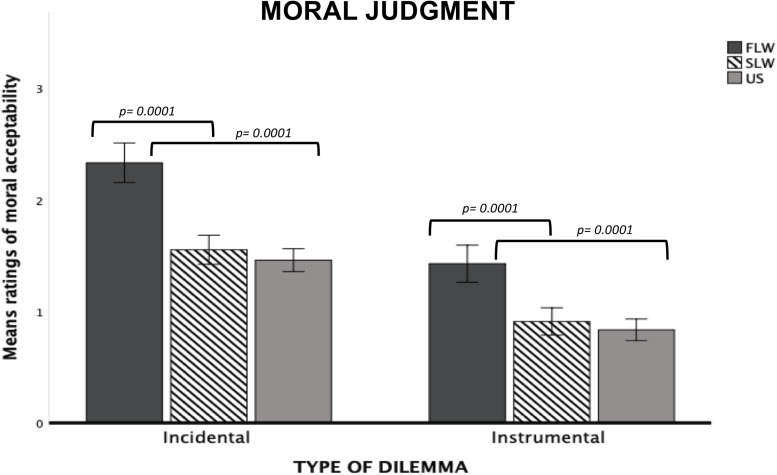
Bar graphs depict the significant Type of Dilemma X Group interaction for mean ratings of moral acceptability in the moral decision-making task. Fisher’s LSD post-hoc comparisons were conducted on significant main effects and interactions. In the figure, statistically significant differences among groups are indicated with *p* values. Error bars indicate the standard errors. FLW, front-line workers; SLW, second-line workers; US, university students.

Finally, we found a significant *Type of Dilemma* × *Group* interaction (F_2,1297_ = 6.91, *p* = 0.001, η${}_{p}^{2}$ = 0.01). In both Incidental and Instrumental dilemmas, the FLW group judged the utilitarian choices as more morally acceptable than the SLW group and the US group; moreover, each group judged the utilitarian choices as more acceptable in Incidental than Instrumental dilemmas (*p* = 0.0001 for each comparison; see Figure 2).

#### Valence

*Type of Dilemma* (*F*_1,1297_ = 571.58, *p* = 0.0001, η${}_{p}^{2}$ = 0.31) and *Risk-involvement* (*F*_1,1297_ = 117.84, *p* = 0.0001, η${}_{p}^{2}$ = 0.08) main effects were both significant. Decision-making during Incidental dilemmas was rated as more unpleasant than during Instrumental dilemmas (*p* = 0.0001); decision-making in Other-involvement dilemmas was rated as more unpleasant compared to Self-involvement dilemmas (*p* = 0.0001). We also observed a significant interaction between *Type of Dilemma* and *Risk-Involvement* (*F*_1,1297_ = 52.60, *p* = 0.0001, η${}_{p}^{2}$ = 0.04). For both Incidental and Instrumental dilemmas, decision-making in Other-involvement dilemmas was rated as more unpleasant than in Self-involvement dilemmas (*p* = 0.0001 for each comparison); moreover, in each risk-involvement condition, decision-making was rated as more unpleasant in Incidental than Instrumental dilemmas.

The significant *Group* main effect (*F*_2,1297_ = 42.45, *p* = 0.0001, η${}_{p}^{2}$ = 0.06) showed that the three groups differed from each others. Specifically, participant in the FLW group reported lower unpleasantness compared to the other two groups (*p* = 0.0001 for each comparisons); on the contrary, the SLW group showed more unpleasantness compared to the FLW group (*p* = 0.0001) and the US group (*p* = 0.002).

**FIGURE 3:**
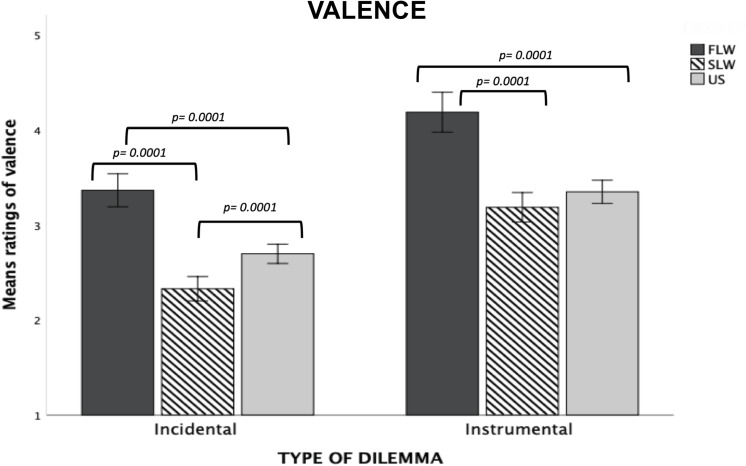
Bar graphs depict the significant Type of Dilemma X Group interaction for mean ratings of valence in the moral decision-making task. Fisher’s LSD post-hoc comparisons were conducted on significant main effects and interactions. In the figure, statistically significant differences among groups are indicated with *p* values. Error bars indicate the standard errors. FLW, front-line workers; SLW, second-line workers; US, university students.

Finally, we found a significant *Type of Dilemma* × *Group* interaction (*F*_2,1297_ = 5.38, *p* = 0.005, η${}_{p}^{2}$ = 0.008). Post-hoc comparisons showed that the three groups differed from each others on Incidental dilemmas. In particular, the FLW reported lower unpleasantness during decision-making than the other two groups (*p* = 0.0001 for each comparison; see Figure 3), on the contrary, the SLW group reported higher unpleasantness as compared to the other groups (*p* = 0.0001 for each comparison); finally, the US group reported higher unpleasantness than the FLW group but lower unpleasantness than SLW group (*p* = 0.0001 for each comparisons). On Instrumental dilemmas, the SLW group reported lower unpleasantness than the FLW group (*p* = 0.0001; see Figure 3); no significant differences were found between the SLW group and the US group.

#### Arousal

*Type of Dilemma* (*F*_1,1297_ = 608.63; *p* = 0.0001, η${}_{p}^{2}$ = 0.32) and *Risk-involvement* (*F*_1,1297_ = 32.72, *p* = 0.0001, η${}_{p}^{2}$ = 0.02) main effects were both significant, with moral decision-making during Incidental dilemmas judged as more arousing than during Instrumental dilemmas (*p* = 0.0001) and killing to save only others as more arousing than killing to save oneself and others (*p* = 0.0001). We also found a significant interaction between the two factors (*F*_1,1297_ = 33.86; *p* = 0.0001, η${}_{p}^{2}$ = 0.02). Post-hoc tests showed that the *Risk-involvement* effect was significant for only Instrumental dilemmas. Specifically, decision-making during Instrumental Other-involvement dilemmas were judged as more arousing than during Instrumental Self-involvement dilemmas (*p* = 0.0001).

The significant *Group* main effect (*F*_2,_1297 = 20.58, p = 0.0001, η${}_{p}^{2}$ = 0.03) showed that the three groups differed from each others. Specifically, the SLW group reported higher arousal than the FLW group (*p* = 0.0001) and the US group (*p* = 0.008). The FLW group showed lower arousal than the US group (*p* = 0.0001).

**FIGURE 4:**
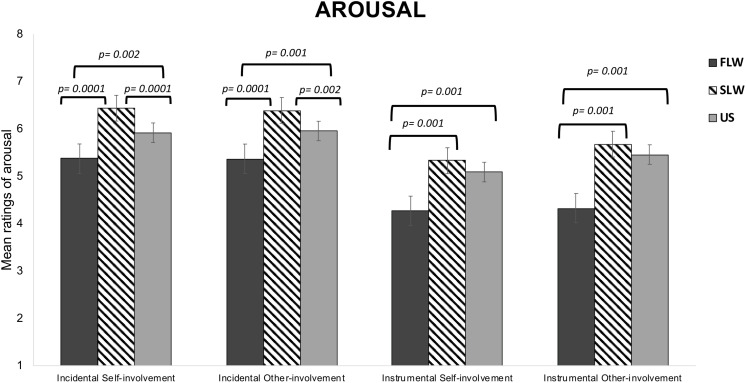
Bar graphs depict the significant Type of Dilemma X Risk-Involvement X Group interaction for mean ratings of arousal in the moral decision-making task. Fisher’s LSD post-hoc comparisons were conducted on significant main effects and interactions. In the figure, statistically significant differences among groups are indicated with *p* values. Error bars indicate the standard errors. FLW, front-line workers; SLW, second-line workers; US, university students.

We also observed significant *Type of Dilemma* × *Group* (*F*_2,1297_ = 12.31, *p* = 0.0001, η${}_{p}^{2}$ = 0.02) and *Risk-involvement* × *Group* (*F*_2,1297_ = 4.88, *p* = 0.008, η${}_{p}^{2}$ = 0.007) interactions. The significant *Type of Dilemma* × *Risk-involvement* × *Group* interaction (*F*_2,1297_ = 4.04, *p* = 0.02, η${}_{p}^{2}$ = 0.006) specified that for Incidental dilemmas the SLW and US groups reported higher arousal in both Other-involvement than Self-involvement scenarios, with no difference in arousal ratings for Instrumental dilemmas; in contrast, the FLW group did not show any significant difference in arousal ratings as a function of risk-involvement; moreover, for Incidental dilemmas, the FLW group reported less arousal as compared to the SLW group and the US group in both risk-involvement conditions, while the group that showed the highest activation was the SLW group. In Instrumental dilemmas, the FLW group showed lower activation as compared to the other two groups, while the SLW and US groups did not differ from each other (see Figure 4).

### Evaluation of Perceived Stress

Our results showed significant differences among the three groups in the PSS scores (*F*_2,1297_ = 97.06, *p* = 0.0001, η${}_{p}^{2}$ = 0.13). Post-hoc comparisons showed that the US group had the highest level of stress, while the FLW group had the lowest level of stress (*p* = 0.0001 for each comparison; see Table 2).

**TABLE 2:** Differences among front-line workers (FLW), second-line workers (SLW), and university students (US) in the scores of the PSS, IRI, and A-ToM scales.

	FLW	SLW	US	*F*_2,1297_	*p*	η${}_{p}^{2}$	LSD post-hoc tests		
	Mean (sd)	Mean (sd)	Mean (sd)						
							*p*		
							FLW *vs* SLW	SLW *vs* US	US *vs* FLW
PSS	12.67 (6.83)	17.40 (6.25)	19.69 (6.65)	97.06	0.0001	0.13	0.0001	0.0001	0.0001
**Empathy measure-IRI**									
PT	20.35 (4.27)	19.10 (4.45)	19.68 (4.82)	5.54	0.004	0.01	0.001	0.04	0.06
FS	15.47 (5.65)	18.02 (5.10)	19.50 (4.63)	56.14	0.0001	0.08	0.0001	0.0001	0.0001
EC	20.21 (4.15)	21.83 (4.30)	21.51 (3.95)	11.85	0.0001	0.02	0.0001	0.23	0.0001
PD	7.13 (5.58)	11.55 (5.88)	12.10 (5.04)	73.03	0.0001	0.10	0.0001	0.11	0.0001
**Theory of Mind measure**									
A-ToM	10.82 (2.26)	10.76 (2.47)	11.67 (1.76)	29.37	0.0001	0.04	0.72	0.0001	0.0001

### Empathy Measure

We found significant differences among the three groups across all IRI subscales: PT (*F*_2,1297_ = 5.54, *p* = 0.004, η${}_{p}^{2}$ = 0.01), FS (*F*_2,1297_ = 56.14, *p* = 0.0001, η${}_{p}^{2}$ = 0.08), EC (*F*_2,1297_ = 11.85, *p* = 0.0001, η${}_{p}^{2}$ = 0.02), PD (*F*_2,1297_ = 73.03, *p* = 0.0001, η${}_{p}^{2}$ = 0.10). Regarding the PT subscale, post-hoc comparisons showed that the FLW group differed from the SLW group, showing more abilities of perspective-taking (*p* = 0.001); in the FS subscale the three groups differed from each others, specifically the US group showed more tendency to identify with fictitious personages (*p* = 0.0001 for each comparison), and the FLW group had lower FS scores than the other two groups (*p* = 0.0001 for each comparison). In the EC and PD subscales, post-hoc comparisons showed that the FLW group had lower scores than the SLW group and the US group (*p* = 0.0001 for each comparison), which did not differ from each other (see Table 2).

### Theory of Mind Measure

We found significant differences among the three groups in the A-ToM scores (*F*_2,1297_ = 29.37, *p* = 0.0001, η${}_{p}^{2}$ = 0.04). Post-hoc comparisons showed that the US group showed a higher mentalizing ability than the other two groups (*p* = 0.0001 for each comparison), which did not differ from each other (see Table 2).

## Discussion

In Study 1 we aimed to compare moral-decision making, level of perceived stress, ability of mentalizing and empathy in Italian workers and university students.

In line with the literature (Sarlo et al., 2012; Lotto et al., 2014; Pletti et al., 2017), we found that participants were more likely to accept utilitarian resolutions and judged these type of choices as more morally acceptable in incidental than instrumental dilemmas. Interestingly, in contrast with what hypothesized by the dual-process model (Greene et al., 2001, 2004, 2008), decision-making in incidental dilemmas was more arousing and more unpleasant than in instrumental dilemmas. Thus, the choice of letting one person die as a foreseen but unintended consequence of saving a larger number of individuals was overall experienced as more emotional, probably because it matched the prototypical feature of the risks people had to face during the COVID-19 pandemic peak.

We also found that our participants, even with showing no differences in utilitarian choices as a function of risk-involvement, judged the act to kill someone as less morally acceptable but less unpleasant and arousing when their own lives were at risk than when they were not at risk. We hypothesize that this result, different from what is suggested by the literature (Lotto et al., 2014; Colangeli et al., 2015), is linked with the fear of contagion that could have influenced moral reasoning. The COVID-19 pandemic highlighted the question of “life and death” and probably made the population more aware of the risk of losing their life due to the contagion and the consequent will to save themselves.

Our findings showed that working condition during COVID-19 seems to affect the moral decision-making ability. Particularly, we found that the FLW participants, compared to the SLW and US groups, were more likely to choose utilitarian responses in both incidental and instrumental dilemmas, regardless of risk involvement. Moreover, the FLW group judged the act of killing one individual to save more lives as more morally acceptable and experienced decision-making as less unpleasant and arousing with respect to the other groups. Contrary to expectations, the FLW group was also less stressed than the other groups. According to Selye (1936), the stress response is characterized by three stages: alarm reaction, resistance and exhaustion. We support the idea that the FLW participants, at the time of our online questionnaire (Italian Phase one of the lockdown), were facing the second stage of the stress response, characterized by the person’s attempt to adapt and cope with the stressor (Selye, 1936; Dias and Neto, 2017). Resilience could be related to an adaptive function of empathy (Williams et al., 2012; Francis et al., 2017). Specifically, a lower level of empathy may promote resilience in emotionally aversive emergencies (Williams et al., 2012; Francis et al., 2017). We found that the FLW group consistently showed lower scores on the PD and EC subscales of IRI. PD refers to personal feelings of anxiety and discomfort that result in observing others’ negative experiences, while EC concerns personal feelings of warmth, compassion and concern for others (Davis, 1980,1983). On the other hand, the FLW group had higher scores on the PT subscale that evaluates spontaneous attempts to cognitively adopt the perspectives of other people and see things from their point of view (Davis, 1980,1983).

Taking together, these findings suggest that, even if the FLW group understands the needs and intentions of people with whom they come into contact, they are able to adopt coping skills and keep emotional regulation. Indeed, reduced emotional reactivity and low-stress levels seem to increase the probability to choose utilitarian judgments (Starcke et al., 2011; Youssef et al., 2012; Sarlo et al., 2014; McNair et al., 2018). This evidence is supported by the results obtained in the moral decision-making task, which highlight a clear utilitarian profile for the FLW group. Indeed, working on the front-line during an emergency, like a pandemic, require more responsibility, more self-control and emotion regulation strategies for own and others’ safety, in the light of a cost-benefit analysis.

On the contrary, the US group showed higher stress levels compared to the other two groups. This finding is in according to other studies suggesting that public health emergencies may increase anxiety, fear and concern in university students (Mei et al., 2011; Cao et al., 2020; Cornine, 2020). Furthermore, the US group had a higher mentalizing ability and were more prone to reject utilitarian resolutions in other-involvement dilemmas, regardless of dilemma type, compared to the FLW group. The US group also tended to judge utilitarian responses as less morally acceptable than the FLW group in incidental dilemmas, regardless of risk-involvement. Moral judgment is the process by which people decide whether an action is correct or wrong, including the evaluation of rights, duties, or obligations (Colby et al., 1980; Tasso et al., 2017). This process of evaluation requires ToM ability to predicting the consequences of our actions and judge how people might react to them (Casebeer, 2003; Baez et al., 2017). We also found that the US group showed higher scores on the FS subscale of IRI. FS is an empathy component that requires the ability to imagine oneself into feeling and actions of characters of books and movies (Davis, 1980). The moral decision-making task explicitly required participants to try to identify themselves with the main character of each scenario (Lotto et al., 2014; Cecchetto et al., 2018; Palmiotti et al., 2020). The higher mentalizing ability and the higher FS scores could explain the greater propensity to adhere to deontological ethical rules in those scenarios in which subjects’ lives were not at risk and when sacrificing one person to save others is only a foreseen but unintended consequence.

As compared to the other two groups, the SLW group showed a lower probability to accept utilitarian resolutions in Instrumental dilemmas, regardless of risk-involvement. According to previous research (e.g., Greene et al., 2001, 2004; Sarlo et al., 2012; Lotto et al., 2014), instrumental dilemmas evoke very strong emotional reactions toward the idea of killing one individual as a means to save others, making participants more likely to support deontological principles. This is in line with the Doctrine of the Double Effect (DDE; Aquinas, 1274/1952), according to which the distinction between the moral intention of a specific action and the consequences of the action itself is fundamental. Specifically, it is morally unacceptable to intentionally kill one individual for a greater good (Manfrinati et al., 2013; Lotto et al., 2014). We found that the SLW was the group that reported overall the highest arousal and unpleasantness during moral decision-making. Moreover, they had higher scores on the EC subscale of IRI, indicating a higher tendency to experience feelings of warmth and compassion toward others. Consistently, previous studies found that the EC scores positively predicted the arousal (Cecchetto et al., 2018) and the unpleasantness (Sarlo et al., 2014; Cecchetto et al., 2018) experienced during the decision-making process in all dilemma types.

The most relevant results in our study concern the FLW group, which was more likely to maximize the overall benefits while maintaining a greater emotional control than the other two groups. This group was composed by workers who were more exposed to contagion risk during the pandemic and by professional categories with absolutely greater responsibility in minimizing the risks and ensuring the safety for other citizens. For this reason, in Study 2 we have decided to analyze the same variables of Study 1 by focusing on the direct comparison between the two subcategories of FLW, i.e., HP and PSP, that during the pandemic have played a key role in emergency management.

## Study 2

The aim of Study 2 was to compare moral decision-making, the level of perceived stress, the ability of mentalizing and empathy in two professional categories, namely healthcare providers (HP) and public safety personnel (PSP), that were particularly engaged in emergency management during COVID-19. The COVID-19 pandemic has put HP around the world facing tough decisions and unprecedented pressure (Greenberg et al., 2020). Specifically, a lack of adequate resources, such as shortage of personnel, lack of beds in Intensive Care Units, ventilators, personal protection equipment hindered the possibility to provide adequate treatment to all patients (Greenberg et al., 2020; Remuzzi and Remuzzi, 2020; Rosenbaum, 2020). Criteria for access to intensive care and discharge based on distributive justice and the appropriate allocation of limited healthcare resources have been defined (Vergano et al., 2020). These criteria establish that intensive treatment must be guaranteed to patients with greater chances of therapeutic success, favoring the “greatest life expectancy” (Vergano et al., 2020). This utilitarian approach can be emotionally burdensome and may cause psychological and moral distress in healthcare providers (Binkley and Kemp, 2020; Greenberg et al., 2020; Prestia, 2020; Williamson et al., 2020).

In addition to the sanitary section, during the COVID-19 pandemic, PSP were called upon to ensure compliance with the restrictive measures established by the Italian Government in order to prevent the transmission of the infection. As pointed out by Pearce et al. (2020) the dynamic nature of the COVID-19 challenge demands that judgments and decisions are made quickly. This principle applies to both HP and PSP as categories of workers most exposed to the risk of infection and with greater decision-making responsibilities.

### Participants

In Study 2, we selected from the total sample (*n* = 1300) 82 HP and 117 PSP. Among these, 13.6% lived in northern regions, 59.8% lived in southern regions and 26.6% lived in central regions. The HP group (mean age = 43.70 years) was composed by doctors, nurses, pharmacists, technicians, therapists, dentists, socio-health workers, Italy’s Red and White Cross volunteers. The PSP group was composed of police officers, carabinieri, army officers, firefighters, and finance guard (mean age = 35.18 years). For details see Table 3.

**TABLE 3:** Differences between healthcare providers (HP) and public safety personnel (PSP) for demographic data.

	HP (*N* = 82)	PSP (*N* = 117)	*F (df)*	*p*	η${}_{p}^{2}$
Mean Chronological Age in years (SD)	43.70 (12.12)	35.18 (8.66)	33.40 (1.197)	0.0001	0.14
Mean Education in years (SD)	18.21 (3.88)	13.15 (1.37)	168.820 (1.197)	0.0001	0.46
Gender (M; F)	38;44	91;26	20.894 (1)*	0.0001*	–

*Chi-square test.

### Statistical Analysis

We performed a one-way ANOVA to evaluate differences between the two groups (HP and PSP) in the sociodemographic data, mean scores of PSS, A-ToM and all subscales of IRI.

Regarding the moral decision-making task, we performed four separate 2 × 2 × 2 repeated-measures ANOVAs on the proportion of utilitarian choices, mean ratings of moral acceptability, means ratings of valence and mean ratings of arousal. For each of these variables, we considered the *Group* (HP and PSP) as a between-subject factor, and *Type of Dilemma* (Incidental *vs* Instrumental) and *Risk-Involvement* (Self- *vs* Other-involvement) as within-subject factors. Fisher’s LSD post-hoc comparisons were conducted on significant main effects and interactions.

The analyses were performed using IBM SPSS Statistics (IBM Corp., 2011).

## Results

### Moral Decision-Making Task

#### Proportion of Utilitarian Choices

**FIGURE 5:**
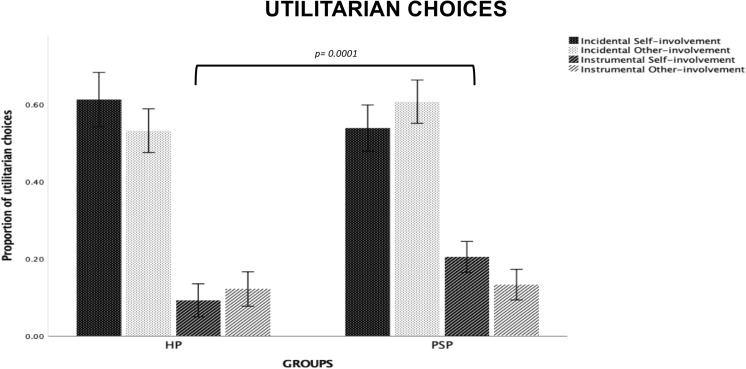
Bar graphs depict the significant Type of Dilemma X Risk-Involvement X Group interaction for proportion of utilitarian choices in the moral decision-making task. Fisher’s LSD post-hoc comparisons were conducted on significant main effects and interactions. In the figure, statistically significant differences between groups are indicated with *p* values. Statistically significant differences within groups are reported in Results section. Error bars indicate the standard errors. HP, healthcare providers (HP); PSP, public safety personnel.

*Type of Dilemma* (*F*_1,197_ = 495.39, *p* = 0.0001, η${}_{p}^{2}$ = 0.71), but not Risk-involvement, was significant, with Incidental dilemmas receiving more utilitarian responses than Instrumental dilemmas (*p* = 0.0001). The *Type of Dilemma* × *Risk-involvement* × *Group* interaction was significant (*F*_1,197_ = 37.37, *p* = 0.0001, η${}_{p}^{2}$ = 0.16) (see Figure 5). Post-hoc tests showed that the PSP group was more likely than HP group to accept utilitarian resolutions on Instrumental Self-involvement dilemmas (*p* = 0.0001); moreover, the PSP group gave a higher number of utilitarian responses in the Self- than in the Other-involvement condition for Instrumental dilemmas, while the opposite was found for Incidental dilemmas; in contrast, the HP group gave a higher number of utilitarian responses in the Self- than in the Other-involvement condition for Incidental dilemmas, with no differences in risk-involvement for Instrumental dilemmas.

#### Moral Judgment

The *Type of Dilemma* (*F*_1,197_ = 151.33, *p* = 0.0001, η${}_{p}^{2}$ = 0.43) and *Risk-involvement* (*F*_1,197_ = 5.99, *p* = 0.01, η${}_{p}^{2}$ = 0.03) main effects were both significant, with the utilitarian choices on Incidental dilemmas and Other-involvement dilemmas judged as more morally acceptable compared to Instrumental dilemmas (*p* = 0.0001) and Self-involvement dilemmas (*p* = 0.01), respectively.

**FIGURE 6:**
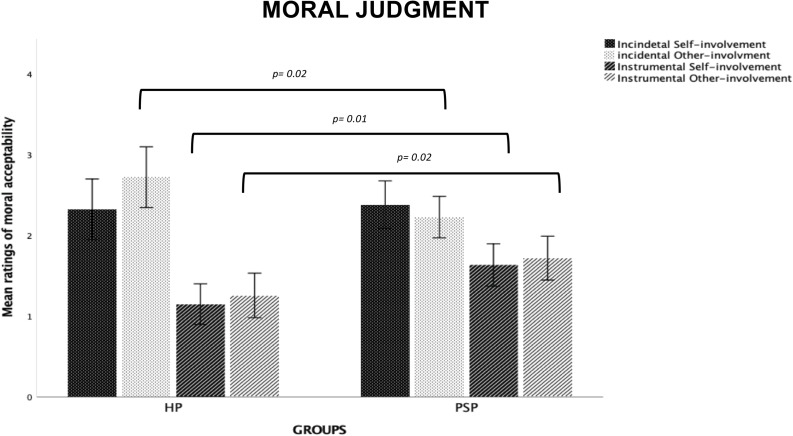
Bar graphs depict the significant Type of Dilemma X Risk-Involvement X Group interaction for mean ratings of moral acceptability in the moral decision-making task. Fisher’s LSD post-hoc comparisons were conducted on significant main effects and interactions. In the figure, statistically significant differences between groups are indicated with *p* values. Error bars indicate the standard errors. HP, healthcare providers (HP); PSP, public safety personnel.

We also found significant *Type of Dilemma* × *Group* (*F*_1,197_ = 19.08, *p* = 0.0001, η${}_{p}^{2}$ = 0.09) and *Risk-involvement* × *Group* (*F*_1,197_ = 10.34, *p* = 0.002, η${}_{p}^{2}$ = 0.05) interactions. The significant *Type of Dilemma* × *Risk-involvement* × *Group* interaction (*F*_1,197_ = 9.95, *p* = 0.002, η${}_{p}^{2}$ = 0.05) specified that the HP group judged utilitarian choices as more morally acceptable than the PSP group on Incidental Other-involvement dilemmas (*p* = 0.02), whereas the PSP group judged utilitarian choices as more morally acceptable than the HP group in both risk-involvement conditions of Instrumental dilemmas (*ps* < 0.03); moreover, the HP group judged utilitarian choices as more acceptable in Incidental Other- than Self-involvement dilemmas, whereas no differences as a function of risk-involvement were found for Instrumental dilemmas or within the PSP group (see Figure 6).

#### Valence

We found a main effect of *Type of Dilemma* (*F*_1,197_ = 74.35, *p* = 0.0001, η${}_{p}^{2}$ = 0.27), with decision-making in Incidental dilemmas rated as more unpleasant than in Instrumental dilemmas (*p* = 0.0001).

**FIGURE 7:**
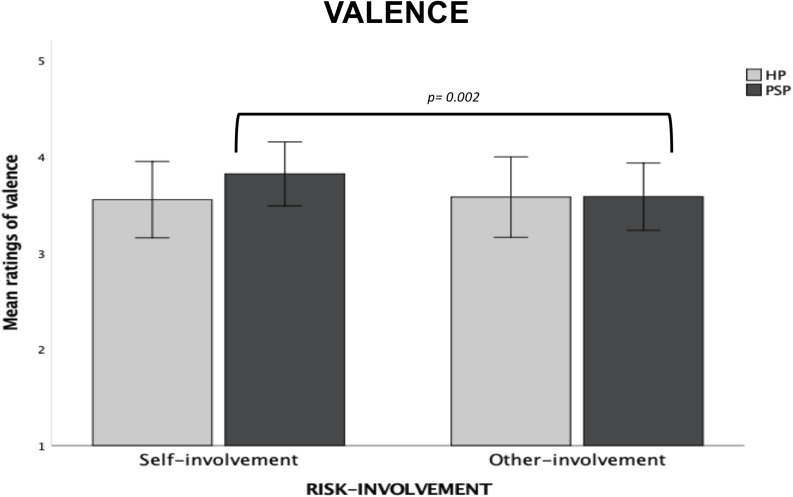
Bar graphs depict the significant Risk-involvement X Group interaction for mean ratings of valence in the moral decision-making task. Fisher’s LSD post-hoc comparisons were conducted on significant main effects and interactions. In the figure, statistically significant differences within groups are indicated with *p* values. No significant differences were found between group. Error bars indicate the standard errors. HP, healthcare providers (HP); PSP, public safety personnel.

We also found a significant *Risk-involvement* × *Group* interaction (*F*_1,197_ = 4.96, *p* = 0.03, η${}_{p}^{2}$ = 0.02). Specifically, *post hoc* analyses showed significant differences only within the PSP group, which rated moral decision-making as more unpleasant when scenarios included killing to save only others (*p* = 0.002). No significant differences were observed between the two groups (see Figure 7).

#### Arousal

The significant *Group* main effect (*F*_1,197_ = 29.99, *p* = 0.0001, η${}_{p}^{2}$ = 0.13) showed that participants in the HP group reported overall more arousal than participants in the PSP group (*p* = 0.0001).

**FIGURE 8:**
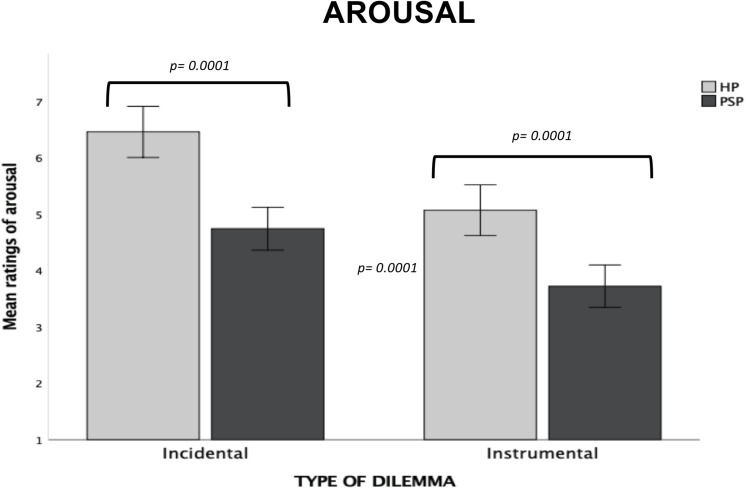
Bar graphs depict the significant Type of Dilemma X Group interaction for mean ratings of arousal in the moral decision-making task. Fisher’s LSD post-hoc comparisons were conducted on significant main effects and interactions. In the figure, statistically significant differences between groups are indicated with *p* values. Error bars indicate the standard errors. HP, healthcare providers (HP); PSP, public safety personnel.

We found a main effect of *Type of Dilemma* (*F*_1,197_ = 177.01, *p* = 0.0001, η${}_{p}^{2}$ = 0.47), with decision-making in Incidental dilemmas receiving higher arousal ratings than in Instrumental dilemmas (*p* = 0.0001). A significant *Type of Dilemma* η${}_{p}^{2}$*Group* interaction was also found (*F*_1,197_ = 4.14, *p* = 0.04, η${}_{p}^{2}$ = 0.02). Specifically, the HP group reported higher arousal than the PSP group during decision-making in both Incidental and Instrumental dilemmas (*p* = 0.0001 for each comparison; see Figure 8); moreover, each group judged the decision-making as more arousing in Incidental than Instrumental dilemmas.

### Evaluation of Perceived Stress

Our results showed significant differences between two groups in the PSS scores (*F*_1,197_ = 7.84, *p* = 0.006, η${}_{p}^{2}$ = 0.04). The HP group had higher level of stress than the PSP group (see Table 4).

**TABLE 4:** Differences between healthcare providers (HP) and public safety personnel (PSP) in the scores of the PSS, IRI, and A-ToM scales.

	FLW	SLW	*F*_2,1297_	*p*	η${}_{p}^{2}$
	Mean (sd)	Mean (sd)			
PSS	14.35 (6.87)	11.70 (6.37)	7.84	0.006	0.038
**Empathy measure-IRI**					
PT	20.06 (4.47)	20.08 (4.06)	0.001	0.98	0.00
FS	16.06 (5.94)	14.41 (5.37)	4.17	0.04	0.02
EC	21.63(3.52)	19.24 (4.19)	17.83	0.0001	0.08
PD	7.32 (5.59)	6.50 (4.91)	1.18	0.28	0.01
**Theory of Mind measure**					
A-ToM	11.01 (2.39)	10.44 (2.28)	3.34	0.07	0.02

### Empathy Measure

The two groups showed significant differences in the FS (*F*_1,197_ = 4.17, *p* = 0.04, η${}_{p}^{2}$ = 0.02) and EC (*F*_1,197_ = 17.83, *p* = 0.0001, η${}_{p}^{2}$ = 0.08) subscales of IRI. Specifically, the HP group reported more tendency to identify themselves with fictitious characters and more feelings of compassion, tenderness, and concern for other people than the PSP group (see Table 4).

### Theory of Mind Measure

We found no significant differences between two groups in the A-ToM scores (see Table 4).

## Discussion

In Study 2 we aimed to compare moral-decision making, level of perceived stress, ability of mentalizing and empathy in two professional categories, namely HP and PSP, that were particularly engaged in emergency management during COVID-19. Both groups, typically, face situations in which they have to make moral decisions as a part of their occupational duties (Murray, 2010; Ransohoff, 2011; Colangeli et al., 2015; Grinberg et al., 2016; Francis et al., 2017).

Overall, as expected, incidental dilemmas elicited a higher proportion of utilitarian responses and were judged as more morally acceptable than instrumental dilemmas. Moreover, moral decision-making in incidental dilemmas was more arousing and more unpleasant than in instrumental dilemmas. Regarding the risk-involvement condition, we found that our participants judged as less morally acceptable killing someone when also their own lives were at risk. This finding is in line with previous studies indicating that killing to save oneself and others is perceived as less morally acceptable than killing to save only others. Thus, sacrificing one individual to save a larger number of people could be perceived as a more virtuous principle when the decision maker’s life is not at risk (Lotto et al., 2014; Colangeli et al., 2015).

In incidental dilemmas, which described killing one individual as a foreseen but unintended consequence of saving others (Lotto et al., 2014), our results demonstrated no significant differences between groups about the choice of action. However, the HP group judged the incidental killing as more morally acceptable than the PSP group when their own lives were not at risk. The goal of utilitarian ethics is to obtain the highest benefits with the lowest cost (Mack, 2004; Mandal et al., 2016; Balducci and Colloca, 2020). This is an approach defined as consequentialist, since the morality of the intervention is determined by the outcomes. It is not surprising that in emergency and extreme situations, such as those described in the moral decision-making task employed in our study, the HP group showed more awareness about the choice to achieve the greatest good for the greatest number of people when the harm is foreseen but unintended, and when their own life is not at risk. In this regard, we found an opposite patterns in the two groups: in incidental dilemmas, the HP group was more likely to accept utilitarian resolution when their own lives were at risk, even if they judged this action as less morally acceptable; on the contrary, the PSP group was more likely to accept the utilitarian resolution when the incidental killing did not include a risk for their own lives. Probably, for the HP group, these results were due to the greater awareness about personal responsibility in guaranteeing the safeguard of other’s people lives. Furthermore, while it is normal for the PSP group to put their lives at risk, for the participants in the HP group this risk occurred with COVID-19 and this could have influenced the cost-benefit analysis. Interestingly, the HP group experienced decision-making as more arousing both in incidental and instrumental dilemmas, regardless of risk-involvement. Arousal reflects a subjective state referring to a sense of mobilization or energy, representing one of the basic components of emotional experience (Lang et al., 1993; Russell, 2003; Duncan and Barrett, 2007) that here is also characterized by high levels of unpleasantness. The HP group reported more intense emotional activation suggesting that solving an ethical-moral problem has a higher emotional cost for them. This is also confirmed by the higher levels of perceived stress and the higher scores in the EC and FS subscales of IRI. In particular, the HP group showed greater empathic concern, which translates into co-participation in the suffering of others. Our results, in line with recent literature on COVID-19, highlights that healthcare workers have been faced enormous pressure during the pandemic, including long working hours, risk of infection, shortages of protective equipment, loneliness, exhaustion, physical fatigue, dealing with patients’ negative emotions and separation from families (Chen et al., 2020; Greenberg et al., 2020; Kang et al., 2020; Liu et al., 2020; Prestia, 2020; Rajkumar, 2020).

In comparison with the HP group, the PSP group showed a greater determination in moral decision-making, indicative of rational thinking, especially in emergencies where decision making determines the sacrifice of few individuals as a means of guaranteeing the safeguard of a greater number of people. Indeed, in instrumental dilemmas the PSP group was more likely to make utilitarian decisions than the HP group, especially in the self-involvement condition, and judged utilitarian responses as more morally acceptable than the HP group, showing a higher level of intentionality and greater adherence to the rules. Overall, during decision-making the PSP reported lower unpleasantness for dilemmas in which their own lives were in danger than for other-involvement dilemmas. We support the idea that these results, taken together, mirror the specific training and experience gained during professional career for the PSP group that requires putting their own life and safety at risk to protect community members and displaying lower empathic engagement and lesser emotional contact with “the others” than the HP group. In fact, even if no differences in mentalizing abilities between two groups were found, the lower levels of stress and empathic concern in the PSP group and the higher arousal overall showed by the HP group during moral decision-making confirm the differences in the subjective evaluation of the emotional experience perceived during decision-making. Our findings are in line with previous studies (Cecchetto et al., 2018) showing that higher arousal ratings were associated with higher scores on the EC subscale of IRI.

This pattern of results is particularly significant if we take into account the specific emergency caused by the COVID-19 pandemic, which required a different psychological, physical and moral commitment from the two groups analyzed. The HP group had to directly face the suffering of patients and their families, often representing the only link between the infected person and the outside world. This duty requires attempting to understand the situation from patients’ point of view, concern for others, and a desire to act to relieve their suffering. On the other hand, the PSP group had the fundamental role of controlling compliance with the quarantine rules and safeguarding the safety of citizens, making choices based on safeguarding the collective good, with a constant focus on a cost-benefit balance.

## Conclusion

In conclusion, in the Study 1, our results show that the workers most exposed to the risk of infection and with a greater burden of responsibility due to their professional roles (the FLW group) are more inclined to act following a utilitarian perspective to achieve the interest of the superior good; furthermore, they are more able to control intense negative emotions when under pressure. In the Study 2, we highlighted that high levels of stress could influence the decision-making of professional categories who carry out work aimed at collective well-being. Indeed, we found that the resolution of moral dilemmas has important emotional involvement for the HP workers, probably due to empathic feelings of concern for suffering others and to a conflict in decisions implying, in any case, adverse consequences in terms of loss of lives.

We have to acknowledge some limitations of our studies. We used an online questionnaire with self-report measures rather than face to face interviews; consequently, study was limited to those with Internet access. Furthermore, participants could not request any clarification on the questions posed and we could not ask any follow-up questions. The measures are entirely self-report and so may be subject to response biases. The online format did not allow us to check some variables such as cognitive functioning, previous history of personal distress, personality characteristics or psychopathological alterations of the participants. Psychopathological characteristics and adverse events may increase vulnerability to stress and could impact on the same biological structures implicated in social cognitive functions (Janiri et al., 2018, 2019). Additionally, we did not collect information on whether participants or their relative/friend contracted the virus.

Another limitation of this study was the snowball sampling strategy to collect data that is not based on a random selection of the sample, so the results could be biased. In addition, we did not collect information on the participation rate. The sample is not representative of all Italian regions; we had an overrepresentation of the central-southern regions compared to those of the north Italy. We are aware that the pandemic has had a more serious impact in the northern regions, thus the extension of the results to the general population could be limited. Future studies should investigate the relations between the experience of the subjects with the pandemic, moral decision -making and social cognition.

Because our study was cross-sectional we cannot infer about temporal relations between variables, so the causal relationships should be interpreted with caution. For reasons of anonymity and confidentiality, we not collected contact details and personal information from the participants. Consequently, we could not conduct a prospective study but only an explorative one.

Finally, our sample presented heterogeneities in socio-demographic characteristics. In Study 1, the US group was younger than the other two groups and, as expected, had lower mean education in years. Furthermore, there were significant differences in sample size and gender distribution among the three groups. These heterogeneities in socio-demographic characteristics depend on the use of an online questionnaire. Thus, we could not match demographic variables among groups. Significant differences in socio-demographic characteristics are also evident in Study 2.

However, even considering these limitations, we believe that the present work might provide useful and timely information to the scientific community since, to the best of our knowledge, no other studies have analyzed moral decision-making and social cognition in Italian workers during the COVID-19 pandemic. Our results highlight the importance of monitoring and safeguarding the psychological and physical health of the professional figures most involved in the fight against COVID-19, in order to prevent moral distress, the development of anxious-depressive symptoms, or post-traumatic stress disorders. We believe that the results of this study could encourage further research to clarify the impact of the health emergency on moral judgments, for example through new experimental paradigms, such as virtual reality, or through follow-up studies that include a specific measure of moral distress.

## Data Availability Statement

The raw data supporting the conclusions of this article will be made available by the authors, without undue reservation.

## Ethics Statement

Ethical review and approval was not required for the study on human participants in accordance with the local legislation and institutional requirements. The patients/participants provided their written informed consent to participate in this study.

## Author Contributions

MM and MA designed the research. MA, MCP, and ST collected the data. MS, MV, and FM analyzed the data. All authors contributed in writing the manuscript.

## Conflict of Interest

The authors declare that the research was conducted in the absence of any commercial or financial relationships that could be construed as a potential conflict of interest.

## Abbreviations

A-ToM: Advanced Theory of Mind Task
EC: empathic concern
FLW: front-line workers
FS: fantasy
HP: healthcare providers
IRI: Interpersonal Reactivity Index
PD: personal distress
PSP: public safety personnel
PSS: Perceived Stress Scale
PT: perspective taking
SLW: second-line workers
ToM: Theory of Mind
US: university students.
